# Effects of Vitamin B12 on the Reductive Transformation of PFOS and F-53B by Nanoscale Zero-Valent Zinc

**DOI:** 10.3390/toxics14100844

**Published:** 2026-09-23

**Authors:** Shunshun Gao, Tao Wang, Yun Yang, Qingzhi Cai, Xiaoxia Lu

**Affiliations:** Ministry of Education Laboratory for Earth Surface Processes, College of Urban and Environmental Sciences, Peking University, Beijing 100871, China

**Keywords:** PFOS, F-53B, Vitamin B12, nanoscale zero-valent zinc, reductive transformation, defluorination

## Abstract

Perfluorooctane sulfonate (PFOS) and 6:2 chlorinated polyfluoroalkyl ether sulfonate (F-53B) are persistent per- and polyfluoroalkyl substances of concern in aquatic environments. This study compared their anaerobic reductive transformation by nanoscale zero-valent zinc (nZn^0^), with and without vitamin B12 (VB12). Solutions containing 1 mg/L PFOS or F-53B were treated with nZn^0^ (278 mg/L) and, where indicated, 0.05 mmol/L VB12 (67.8 mg/L) at pH 8.5 and 30 °C for 20 days. After correction for losses observed in reagent-free blanks, PFOS loss was negligible with nZn^0^ alone but reached approximately 34% with nZn^0^ + VB12, and the corresponding defluorination rate was 33.9%. In contrast, F-53B showed approximately 85% blank-corrected loss by day 6 with either nZn^0^ alone or nZn^0^ + VB12, while defluorination rates reached 38.0% and 30.7%, respectively, indicating no enhancing benefit of VB12 for F-53B under the studied conditions. Nominal-mass LC-MS/MS features consistent with partially defluorinated and hydrogen-substituted products were observed, and tentative transformation pathways were proposed. These results demonstrated compound-specific effects of VB12 and emphasized the distinction among loss, transformation and defluorination when evaluating PFAS destruction.

## 1. Introduction

Perfluorooctane sulfonate (PFOS) is a legacy per- and polyfluoroalkyl substance (PFAS) that has been used in industrial applications, aqueous film-forming foams, and consumer products. Its exceptional persistence and bioaccumulation potential have led to widespread environmental and health concerns [1,2,3,4]. In 2009, PFOS and related compounds were listed under the Stockholm Convention on Persistent Organic Pollutants. More recently, the International Agency for Research on Cancer classified PFOS as possibly carcinogenic to humans (Group 2B), underscoring the need to limit exposure and develop effective destruction technologies [5].

Following restrictions on PFOS, 6:2 chlorinated polyfluoroalkyl ether sulfonate (F-53B; 6:2 Cl-PFESA) has been used extensively as a chromium-mist suppressant in the Chinese electroplating industry. Industrial releases have resulted in its detection in water, soil, sediment, and biota, and experimental studies have reported biological effects that warrant continued toxicological attention [6,7,8,9,10,11]. Because F-53B is highly persistent and structurally distinct from PFOS, its environmental transformation cannot be assumed to follow the same pathways as those of legacy perfluoroalkyl sulfonates.

PFOS and F-53B are both difficult to destroy under conventional environmental conditions, and their different molecular structures may confer markedly different reductive reactivities. PFOS contains a fully fluorinated C8 chain and a sulfonate headgroup, whereas F-53B contains a chlorine substituent and an ether linkage that can provide comparatively labile reaction sites.

Water-treatment approaches for PFAS include separation technologies, such as adsorption and membrane processes, and destructive technologies based on electrochemical, photochemical, plasma, thermal, or reductive chemistry [12]. Separation can efficiently lower aqueous concentrations but transfers PFAS to a secondary waste stream. Destructive treatment aims to cleave strong C-F bonds, but many approaches require substantial energy or specialized reaction conditions. Biological defluorination of PFOS/PFOA has also been reported under specialized Feammox conditions [13], although biological destruction remains comparatively slow and system-dependent.

For F-53B, adsorption by activated carbon, anion-exchange resins, and layered double hydroxides has shown high removal capacity [14,15,16], whereas destructive treatment by UV/iodide reduction, electrochemical oxidation, and plasma discharge can achieve extensive parent-compound loss and partial to high defluorination under optimized conditions [17,18,19]. These studies also indicate that removal of the parent compound and cleavage of C-F bonds are distinct endpoints and should be evaluated separately.

Vitamin B12 (VB12) contains a redox-active cobalt center and has been used with low-valent metals to mediate reductive transformation of fluorinated compounds. Previous work showed that VB12 combined with reductants can transform selected perfluoroalkyl sulfonates, while the efficiency depends strongly on molecular structure and reductant chemistry [20,21]. However, PFOS and F-53B have rarely been compared side-by-side under identical nZn^0^-based anaerobic conditions. Therefore, this study compared parent-compound loss, fluoride release, and putative transformation products of PFOS and F-53B in three systems: reagent-free blank, nZn^0^ alone, and nZn^0^ + VB12. Particular emphasis was placed on distinguishing apparent parent loss from defluorination and on determining whether VB12 provides a measurable enhancing benefit for each compound.

## 2. Materials and Methods

### 2.1. Chemicals

PFOS (purity ≥99%) and F-53B (purity ≥97%) were purchased from AccuStandard (New Haven, CT, USA). Methanol (HPLC grade) and ammonium acetate (Analytical grade) were purchased from Beijing Tongguang Fine Chemical Company (Beijing, China). Vitamin B12(VB12) (purity ≥99%) was purchased from Beijing Konoscience Technology Ltd. (Beijing, China). Nanoscale zero-valent zinc (nZn^0^) (purity ≥99.9%) was purchased from Shanghai Aladdin Biochemical Technology Co., Ltd. (Shanghai, China).

### 2.2. Experimental Setup

For each target compound, three treatment conditions were established to evaluate reductive transformation: nZn^0^ + VB12, nZn^0^-alone, and a reagent-free Blank (Blank). All experiments were performed in triplicate. The three replicates represent independent experimental reactors (vials), not repeated measurements from the same reactor. The comparison between the nZn^0^ + VB12 and nZn^0^-alone treatments was used to assess the incremental effect of VB12 in the presence of nZn^0^; a VB12-only treatment was not included. Table 1 shows the setup conditions for the experiments.

For the experiments, stock solutions were mixed to obtain 1 mg/L PFOS or F-53B and 1 mg/L resazurin (as a redox indicator), and the pH was adjusted to approximately 8.5. The nZn^0^ + VB12 treatment additionally contained 67.8 mg/L VB12 (approximately 0.05 mmol/L). Five milligrams of nZn^0^ were added to each 20 mL glass headspace vial used for the nZn^0^ + VB12 and nZn^0^-alone treatments; no zinc was added to the Blank. After 18 mL of solution was added, the nominal nZn^0^ loading was about 278 mg/L. Vials were prepared in an anaerobic glove box, purged with N_2_ for approximately 1 h, crimp-sealed with PTFE-faced septa, and incubated at 30 °C and 120 rpm for 20 days. The anaerobic/reducing conditions were qualitatively monitored using resazurin, which remained colorless throughout the reaction, indicating that reducing conditions were maintained; no direct measurements of dissolved oxygen (DO) or redox potential (Eh) were performed. The choices of the initial concentrations were based on the literature [21]. These conditions were selected primarily for laboratory/mechanistic investigation.

Vials were destructively sampled on days 0, 6, and 20. Samples were filtered through 0.45 μm polyethersulfone (PES) syringe filters (Beijing Hongbo Technology Co., Ltd., Beijing, China). One aliquot was analyzed promptly for fluoride using an ion-selective electrode, while the second aliquot was stored at −20 °C and subsequently analyzed by LC-MS/MS. Blank samples were processed identically to the reactive treatments to quantify non-reactive parent-compound losses during handling and incubation.

### 2.3. Sample Pretreatment Methods

Two sample-preparation approaches, solid-phase extraction (SPE) and direct filtration, were evaluated for LC-MS/MS analysis.

In the SPE method, WAX SPE cartridges (150 mg, 6 mL, J&K Scientific, Beijing, China) were used for sample purification. Samples were first filtered through 0.45 μm PES syringe filters to remove solid particles. The cartridges were sequentially conditioned with 4 mL of methanol and 4 mL of methanol containing 0.5% ammonium hydroxide, and equilibrated with 4 mL of deionized water. After loading 1 mL of sample, the cartridges were rinsed with 4 mL of 25 mM ammonium acetate (pH = 4), dried under vacuum for 15 min, and eluted with 3 mL of methanol and 3 mL of methanol containing 0.5% ammonium hydroxide. The eluate was evaporated to dryness under nitrogen and reconstituted in 1 mL of deionized water. The flow rate throughout the SPE procedure was maintained below 5 mL/min, and the reconstituted solution was filtered through 0.22 μm PES syringe filters prior to analysis.

In the direct filtration method, samples were directly filtered through 0.22 μm PES syringe filters and transferred to autosampler vials. Recovery of the direct-filtration procedure was evaluated using three replicate samples at 1 mg/L. The measured recoveries of both PFOS and F-53B exceeded 95%.

As shown in Figure S1 in the Supplementary Materials, the two pretreatment approaches produced comparable LC-MS/MS responses under the tested conditions. Because direct filtration was simpler and recovery exceeded 95% for both PFOS and F-53B, direct filtration was used for subsequent samples.

In addition, procedural blank samples were included in this study and were subjected to exactly the same treatment and filtration procedures as the actual reaction samples, in order to monitor potential background contamination introduced throughout the entire analytical workflow. No significant signals of PFOS or F-53B were detected in the blanks, indicating the absence of notable background contamination in the experimental procedure.

### 2.4. Analysis of PFOS, F-53B, and Putative Transformation Products

Two LC-MS/MS systems were used: a Waters ACQUITY UPLC coupled to a Waters XEVO TQ-XS triple-quadrupole mass spectrometer (Waters, Milford, MA, USA) equipped with an ACQUITY UPLC BEH C_18_ column (2.1 mm × 100 mm, 1.7 μm, Waters, Milford, MA, USA) for the analysis of PFOS; and an AB Sciex API 4000 (AB Sciex, Framingham, MA, USA) equipped with an Agilent InfinityLab Poroshell 120 EC-C_18_ column (2.1 mm × 100 mm, 2.7 μm, Framingham, MA, USA) for the quantification of F-53B. Multiple-reaction monitoring (MRM) was used to quantify PFOS and F-53B. Full-scan data were used only to tentatively assign product-related signals on the basis of nominal *m*/*z* values and previously reported pathways; these assignments do not constitute confirmed structural identifications because authentic standards and high-resolution MS/MS confirmation were not available [22].

The operating parameters for the Waters ACQUITY UPLC were as follows: column temperature 50 °C; sample chamber temperature 8 °C; flow rate 0.25 mL/min; injection volume 2 μL; mobile phases consisting of 20 mM ammonium acetate in water (A) and methanol (B), with gradient elution as follows: 15% B from 0 to 1.00 min, ramped to 20% B at 1.00 min, to 45% B at 3.00 min, to 80% B at 7.50 min, then to 95% B at 9.00 min, held at 95% B until 10.90 min, returned to 15% B at 11.00 min, and held until 13.00 min for re-equilibration. The autosampler rinse solution was acetonitrile/water (9:1, *v*/*v*), with a rinse time of 6.0 s both before and after injection. The Waters XEVO TQ-XS mass spectrometer was operated in negative-ion ESI mode; the full-scan range was *m*/*z* 50–600, with a scan time of 0.500 s/cycle. Under the above conditions, the retention time of the PFOS standard was 8.20 min.

The operating parameters for the AB Sciex API 4000 were as follows: column temperature 35 °C; flow rate 0.3 mL/min; injection volume 2 μL; mobile phases consisting of 10 mM ammonium acetate in water (A) and methanol (B), with isocratic elution at 70% B (30% A) from 0 to 2.50 min. The autosampler rinse volume was 200 μL, with a “wash before and after aspiration” mode. The mass spectrometer was operated in negative-ion electrospray ionization (ESI) mode; the Q1 full-scan range was *m*/*z* 50–800, with a scan time of 1.00 s/cycle and a step size of 0.10 Da. Under the above conditions, the retention time of the F-53B standard was 1.80 min.

PFOS and F-53B were quantified using external calibration. For PFOS, four calibration levels (100, 250, 500, and 1000 μg/L) were used; for F-53B, five levels (10, 100, 250, 500, and 1000 μg/L) were used. Calibration curves were generated by linear regression of chromatographic response versus concentration. The calibration curve for PFOS over the range of 100–1000 μg/L had an R^2^ of 0.9982, and that for F-53B over the range of 10–1000 μg/L had an R^2^ of 0.9973. Both R^2^ values exceeded 0.997, indicating good linearity of the calibration curves. The limit of detection (LOD) and limit of quantification (LOQ) for PFOS were 0.022 ng/L and 0.073 ng/L, respectively. The LOD and LOQ for F-53B were 0.020 and 0.067 ng/L, respectively.

To evaluate the influence of solvent/matrix differences on quantification, cultured samples were analyzed in preliminary experiments using both pure water and a 7:3 (*v*/*v*) methanol/water system. The results obtained under the two solvent systems were consistent, indicating that, within the concentration range of this study, solvent system differences had little effect on the quantification of PFOS and F-53B, supporting the rationale for using external calibration.

Putative transformation products were screened by comparing full-scan chromatographic features at nominal *m*/*z* values with formulas and pathways reported previously for PFOS or F-53B. Because authentic standards, accurate-mass measurements, and diagnostic MS/MS spectra were not available for these tentative products, the reported formulas and structures are considered tentative assignments rather than confirmed identifications [22]. Temporal changes were therefore described using relative signal intensity or peak area for the same feature, not absolute concentrations or direct quantitative comparisons among different product ions.

Observed parent-compound removal rate was calculated for each treatment from the measured decrease in PFOS or F-53B concentration according to Equation (1):

(1) $$\mathrm{Removal}\;\mathrm{rate}\;(\%)\;=\frac{C_{0}-C_{t}}{C_{0}}\times 100\%$$

In Equation (1), $C_{0}$ was the initial concentration of PFOS and F-53B (1 mg/L), and $C_{t}$ was the corresponding concentration at day *t*.

The blank-corrected removal rate was calculated by accounting for the parent-compound losses observed in the reagent-free Blank according to Equation (2):

(2) $$\mathrm{Blank-corrected}\;\mathrm{removal}\;\mathrm{rate}\;(\%)\;=(\frac{C_{0}^{{nZn}^{0}}-C_{t}^{{nZn}^{0}}}{C_{0}^{{nZn}^{0}}}-\frac{C_{0}^{blank}-C_{t}^{blank}}{C_{0}^{blank}})\times 100\%$$

In Equation (2), $C_{0}$ was the initial concentration of PFOS or F-53B in the nZn^0^ + VB12 or nZn^0^-alone treatment, $C_{0}^{blank}$ was the initial concentration of PFOS or F-53B in the Blank; $C_{t}$ was the concentration of PFOS or F-53B in the nZn^0^ + VB12 or nZn^0^-alone treatment at day *t*, $C_{0}^{blank}$ was the concentration of PFOS or F-53B in the Blank at day *t*.

It should be noted that the blank correction in this study only accounted for non-reactive losses associated with vials, septa, filtration, and handling, but did not correct for PFOS or F-53B sorption onto the nZn^0^ particle surfaces. Therefore, the blank-corrected removal rates should be interpreted as apparent removal, which may include both sorption onto nZn^0^ and chemical transformation.

### 2.5. Analysis of Fluoride Ions and Defluorination

To compare the accuracy of ion chromatography (IC) and fluoride ion selective electrode (ISE) for fluoride quantification, a series of fluoride standard solutions at concentrations of 5, 10, 20, 40, 100 µg/L and 1, 5, 10, 15, 25 mg/L were prepared and measured by both methods.

For IC analysis, samples were first filtered through 0.45 μm PES syringe filters. C18 cartridges were conditioned with 5 mL of methanol and 10 mL of ultrapure water, and H cartridges were conditioned with 20 mL of ultrapure water, followed by equilibration for 20 min. During loading, 5 mL of sample was first passed through the C18 cartridge and discarded, after which the remaining sample was loaded, with the effluent subsequently passing through the H cartridge. The flow rate was maintained below approximately 2 mL/min throughout, and the final effluent was filtered through 0.22 μm PES syringe filters prior to IC analysis.

For ISE measurement, samples were filtered through 0.22 μm PES syringe filters, without the addition of total ionic strength adjustment buffer (TISAB) or additional pH adjustment. The electrode was calibrated with fluoride standards at 10, 100, 1000, and 10,000 µg/L prior to direct sample measurement.

The two fluoride methods showed agreement within approximately 10% over the tested standards (Figure S2 in the Supplementary Materials); the ISE method was therefore used for subsequent samples. It should be noted that this validation supports instrumental consistency under standard conditions, while matrix-related uncertainty in the reaction samples remains.

Defluorination rates for PFOS and F-53B were calculated from blank-corrected fluoride release according to Equations (3) and (4):

(3) $$\mathrm{PFOS}\;\mathrm{defluorination}\;\mathrm{rate}\;(\%)\;=\frac{C_{t}^{F}-C_{t-blank}^{F}}{C_{0}\times 17}\times \frac{M_{PFOS}}{M_{F}}\times 100\%$$

(4) $$F\text{-}53B\;\mathrm{defluorination}\;\mathrm{rate}\;(\%)=\frac{C_{t}^{F}-C_{t-blank}^{F}}{C_{0}\times 16}\times \frac{M_{F-53B}}{M_{F}}\times 100\%$$

In Equation (3), $C_{t}^{F}$ was the fluoride ion concentration (µg/L) in the nZn^0^ + VB12 and nZn^0^-alone treatments at day *t*, $C_{t-blank}^{F}$ was the fluoride ion concentration in the Blank at day *t*, $\;C_{0}$ was the initial concentration of PFOS (1 mg/L), 17 was the number of fluorine atoms in the PFOS molecule, $M_{PFOS}$ was the relative molecular mass of PFOS, and $M_{F}$ was the relative atomic mass of fluorine.

In Equation (4), $C_{t}^{F}$ was the fluoride ion concentration (µg/L) in the nZn^0^ + VB12 and nZn^0^-alone treatments at day t, $C_{t-blank}^{F}$ was the fluoride ion concentration in the Blank at day t, $C_{0}$ was the initial concentration of F-53B (1 mg/L), 16 was the number of fluorine atoms in the F-53B molecule, $M_{F-53B}$ was the relative molecular mass of F-53B, and $M_{F}$ was the relative atomic mass of fluorine.

### 2.6. Statistical Analysis

All experiments were performed in triplicate, and *t*-test and Welch’s *t*-test (two-tailed) were used to compare the differences between treatment groups at the same time points, with the significance level set at *p* < 0.05.

## 3. Results and Discussion

### 3.1. PFOS Transformation and Defluorination

The changes in the concentration, removal rate, and blank-corrected removal rate of PFOS, the concentration of fluoride ion, and the defluorination rate of PFOS over time were shown in Figure 1.

PFOS removal in the reagent-free Blank increased from approximately 8.5% at day 0 to about 24% at day 20. Given the persistence of PFOS and the absence of reactive reagents, this loss was more plausibly attributed to non-reactive processes such as sorption to vial components, filters, septa, or other laboratory surfaces and to handling losses rather than transformation. Sorption of PFAS to polymeric materials and time-dependent losses during controlled laboratory handling have been documented [23,24,25]. The Blank was therefore used to correct treatment-associated parent loss, while recognizing that the correction does not distinguish sorption from chemical transformation within the reactive treatments.

In the nZn^0^ + VB12 treatment, the observed PFOS removal rate reached approximately 58% by day 20, corresponding to approximately 34% blank-corrected loss. By contrast, the nZn^0^-alone treatment showed little blank-corrected PFOS loss. Fluoride release in the nZn^0^ + VB12 treatment increased over the 20-day incubation, with little detectable release before day 6 and a stronger increase thereafter. The average fluoride generation rate over 20 days was 10.95 μg/(L·d), and the PFOS defluorination rate reached approximately 34%. No measurable fluoride release was observed in the nZn^0^-alone or Blank treatment.

At day 20, the PFOS concentration in the nZn^0^ + VB12 treatment (0.420 ± 0.070 mg/L, mean ± SD) was significantly lower than that in the nZn^0^-alone treatment (0.765 ± 0.044 mg/L) (t = 7.25, df = 4, *p* = 0.0019, *t*-test), indicating that VB12 significantly enhanced PFOS transformation in the presence of nZn^0^.

The blank-corrected removal rate and defluorination rate were two independent endpoints supporting enhanced reductive transformation of PFOS when VB12 was present with nZn^0^ under the studied condition. Because a VB12-only treatment was not included, the result should be interpreted as a VB12-associated enhancement relative to nZn^0^ alone, rather than as evidence that VB12 alone was sufficient for transformation.

Five nominal-mass LC-MS features were detected as tentative transformation products during the 20-day treatment period and were tentatively assigned to C_8_HF_16_SO_3_^−^ (*m*/*z* 481), C_8_H_4_F_11_SO_3_^−^ (*m*/*z* 389), C_8_H_5_F_10_SO_3_^−^ (*m*/*z* 371), C_8_H_6_F_9_SO_3_^−^ (*m*/*z* 353), and C_8_H_6_F_7_SO_3_^−^ (*m*/*z* 315). None of these tentative transformation products were detected at day 0. At day 6, the detected products were *m*/*z* 481, 389, 371, and 353 features, whereas at day 20, *m*/*z* 389, 353, and 315 features were detected, as shown in Figure 2. The *m*/*z* 481 and 371 features were observed only on day 6 and had comparatively weak signals. Features at *m*/*z* 389 and 353 were observed on days 6 and 20; the *m*/*z* 389 signal decreased while the *m*/*z* 353 signal increased over time. The *m*/*z* 315 feature appeared only on day 20 and had a comparatively strong response. These temporal patterns were consistent with sequential conversion, but they did not by themselves establish precursor–product relationships because the intermediates were not quantified with authentic standards. Relative temporal trends were assessed from the signal intensity/peak area of each feature. Since these assignments were based on the nominal *m*/*z* and literature pathways, the structures should be regarded as putative [22].

A tentative PFOS transformation pathway was proposed based on the temporal signal patterns in this study and the VB12-mediated PFOS transformation reported by Park et al. [20], as shown in Figure 3. The observed features were compatible with progressive replacement of fluorine by hydrogen and further defluorination. The *m*/*z* 481 feature represented an early monohydrogen-substituted product, while the *m*/*z* 389, 371, 353, and 315 features represented increasingly transformed products. The proposed sequence was one possible interpretation consistent with the observed features and the previous literature, but the exact structures and reaction order require confirmation by authentic standards [22].

### 3.2. F-53B Transformation and Defluorination

The changes in the concentration, removal rate, and blank-corrected removal rate of F-53B, the concentration of fluoride ion, and the defluorination rate of F-53B over time were shown in Figure 4.

The removal rate of F-53B in the reagent-free Blank increased from approximately 13% at day 0 to about 15% at day 20, likely due to non-reactive loss during incubation and sample handling [24]. In both the nZn^0^ + VB12 and nZn^0^-alone treatments, approximately 85% blank-corrected F-53B removal rates were obtained at day 6, with little additional change thereafter. Fluoride release increased in both the nZn^0^ + VB12 and nZn^0^-alone treatments. By day 20, the fluoride generation rates were 8.18 μg/(L·d) for nZn^0^ + VB12 and 10.13 μg/(L·d) for nZn^0^-alone, corresponding to F-53B defluorination rates of approximately 31% and 38%, respectively. The temporary decrease in measured free fluoride in the nZn^0^-alone treatment may reflect fluoride sequestration by Zn^2+^, including possible ZnF_2_ formation; if so, fluoride-based defluorination would underestimate total C-F cleavage. This possibility was not directly quantified and should be treated as a mechanistic explanation rather than a confirmed mass-balance result.

At day 6, due to the damage of one sample vial during incubation, the F-53B data for the nZn^0^ + VB12 treatment were based on two valid replicates. Statistical analysis showed no significant difference between the two treatments at this time point (Welch’s *t*-test, t = 0.893, df = 1, *p* = 0.535). At day 20, the difference between the two treatments was also not statistically significant (Welch‘s *t*-test, t = 3.41, df = 2, *p* = 0.0765). These results indicate that VB12 did not significantly enhance F-53B transformation under the studied conditions.

Seven nominal-mass LC-MS features were tentatively assigned to F-53B-related transformation products: HC_6_F_12_OC_2_F_4_SO_3_^−^ (*m*/*z* 497), HC_5_F_10_OC_2_F_4_SO_3_^−^ (*m*/*z* 447), HC_2_F_4_OC_2_F_4_SO_3_^−^ (*m*/*z* 297), HCF_2_OC_2_F_4_SO_3_^−^ (*m*/*z* 247), HOC_2_F_4_SO_3_^−^ (*m*/*z* 197), HC_2_F_4_SO_3_^−^ (*m*/*z* 181), and a feature assigned as −OOCCF_2_SO_3_^−^ (*m*/*z* 174), as shown in Figure 5. Because authentic standards and high-resolution MS/MS confirmation were unavailable, these formulas and structures are putative [22]. Relative trends were explored using signal intensity/peak area rather than absolute intermediate concentrations, as shown in Figure 5.

The *m*/*z* 497 feature occurred in the nZn^0^ + VB12, nZn^0^-alone, and Blank treatments and therefore could not by itself confirm a treatment-generated dechlorination product. Its approximately stable signal in the Blank but increased signal in the reactive treatments was nevertheless consistent with additional formation during reaction, in agreement with hydrogen-substituted F-53B species previously reported near metal-plating sources [26]. The other features showed treatment- and time-dependent patterns: *m*/*z* 447 and 297 were comparatively persistent, whereas the *m*/*z* 247 signal decreased as the *m*/*z* 197 and 181 signals increased in the nZn^0^ + VB12 treatment. The *m*/*z* 174 feature increased with time. These patterns supported, but did not prove, sequential dechlorination/defluorination and chain-shortening reactions.

Combining the observed nominal-mass features with prior F-53B transformation studies [17,26], a tentative F-53B transformation pathway was proposed in Figure 6. A possible first step was consistent with dechlorination/hydrogen substitution to form a species with nominal *m*/*z* 497, possibly followed by formation of lower-mass fluorinated species. Differences of approximately 50 Da among several assigned features were consistent with loss of CF_2_ units. However, the presence of the *m*/*z* 497 feature in the Blank and the lack of authentic standards precluded definitive structural or precursor–product assignments. The proposed pathway should therefore be considered a working hypothesis for subsequent confirmation [22].

### 3.3. Limitations and Future Work

Several design and analytical constraints affect interpretation.

First, the present study did not include a VB12-only treatment, nor did it quantitatively analyze the concentration changes in VB12 in the solution after the reaction. Therefore, it is not possible to determine whether VB12 is regenerated or consumed during the reaction, nor can a catalytic role of VB12 be established. Accordingly, the role of VB12 in this study should be understood as an “enhancement” relative to nZn^0^ alone, rather than a demonstrated “catalytic” effect.

Second, tentative transformation products were assigned from nominal-mass full-scan data without authentic standards, so the proposed structures and pathways were tentative [22].

Third, fluoride release did not provide a complete fluorine mass balance and might be affected by fluoride sequestration/precipitation in zinc-containing systems. Because Zn^2+^ could bind or precipitate fluoride, particularly as ZnF_2_, ISE-based fluoride release should be interpreted as measured free fluoride and might underestimate total defluorination in zinc-containing systems. The calculated defluorination efficiency should be regarded as an estimate based on free fluoride content, and not as a comprehensive indicator of C–F bond scission.

Fourth, for the analyses of PFOS and F-53B, replicate analytical precision (RSD) was not separately determined, although triplicate replicates of reactors were performed at each time point. External calibration was used for the reported samples without isotope-labelled internal standards. It should be noted that high R^2^ values (>0.997) and recovery (>95%) alone do not demonstrate the absence of matrix effects in the actual reaction samples, and matrix effects may still affect the accuracy of absolute quantification.

It should be emphasized that all removal rates reported in this study may include contributions from both sorption and chemical transformation. Future studies should distinguish sorption from transformation by analyzing PFAS associated with the nZn^0^ solid phase.

Future work should also confirm transformation products by authentic standards where available, close the fluorine mass balance using total or extractable organofluorine measurements, quantify fluoride partitioning/precipitation in zinc-containing systems, evaluate the toxicity of treated solutions and individual transformation products, test performance in environmentally relevant matrices and concentrations, and test the reutilization of these materials for reactions. Such studies are needed before the nZn^0^/VB12 approach can be evaluated as a practical PFAS destruction technology.

As a prospective direction for future research, the nZn^0^/VB12 system might be explored for application in permeable reactive barriers (PRB) for treating PFAS-contaminated groundwater, where nZn^0^ and VB12 could serve as filling materials and treatment would occur as groundwater flows through the barrier. This concept remains speculative and requires substantial further validation.

## 4. Conclusions

This study compared the anaerobic reductive transformation of PFOS and F-53B by nZn^0^ with and without VB12. The two PFAS showed clearly different responses to VB12 and to the nZn^0^-based reductive environment.

(1) PFOS showed little blank-corrected loss and defluorination with nZn^0^ alone, whereas the nZn^0^ + VB12 treatment produced approximately 34% blank-corrected removal rate and 33.9% defluorination rate by day 20. Thus, VB12 enhanced PFOS transformation in the presence of nZn^0^. F-53B behaved differently: approximately 85% blank-corrected loss was reached by day 6 both with and without VB12, and the defluorination rate was 30.7% with nZn^0^ + VB12 versus 38.0% with nZn^0^ alone. VB12 therefore did not enhance F-53B transformation under the studied conditions. Statistical analysis showed that VB12 significantly enhanced PFOS transformation at day 20 (*p* = 0.0019), whereas no significant enhancement was observed for F-53B at either day 6 (*p* = 0.535) or day 20 (*p* = 0.0765). These results indicate that the effect of VB12 is compound-specific.
(2) Fluoride release provided direct evidence of C-F bond cleavage, while nominal-mass LC-MS/MS features were consistent with partially defluorinated and tentative hydrogen-substituted products. Because product assignments were made without authentic standards, the proposed PFOS and F-53B pathways were tentative rather than definitive. The results nevertheless supported structure-dependent reductive reactivity, with the chlorine substituent and ether linkage in F-53B plausibly contributing to its higher reactivity relative to PFOS.
(3) Neither treatment achieved complete defluorination, and the remaining organofluorine signals indicated that the treated solutions could not be assumed to be fully mineralized or detoxified. In addition, parent-compound loss was an operational endpoint that could include sorption as well as transformation. These distinctions are particularly important for remediation studies, where apparent removal alone may overestimate destruction performance.

## Figures and Tables

**Figure 1:**
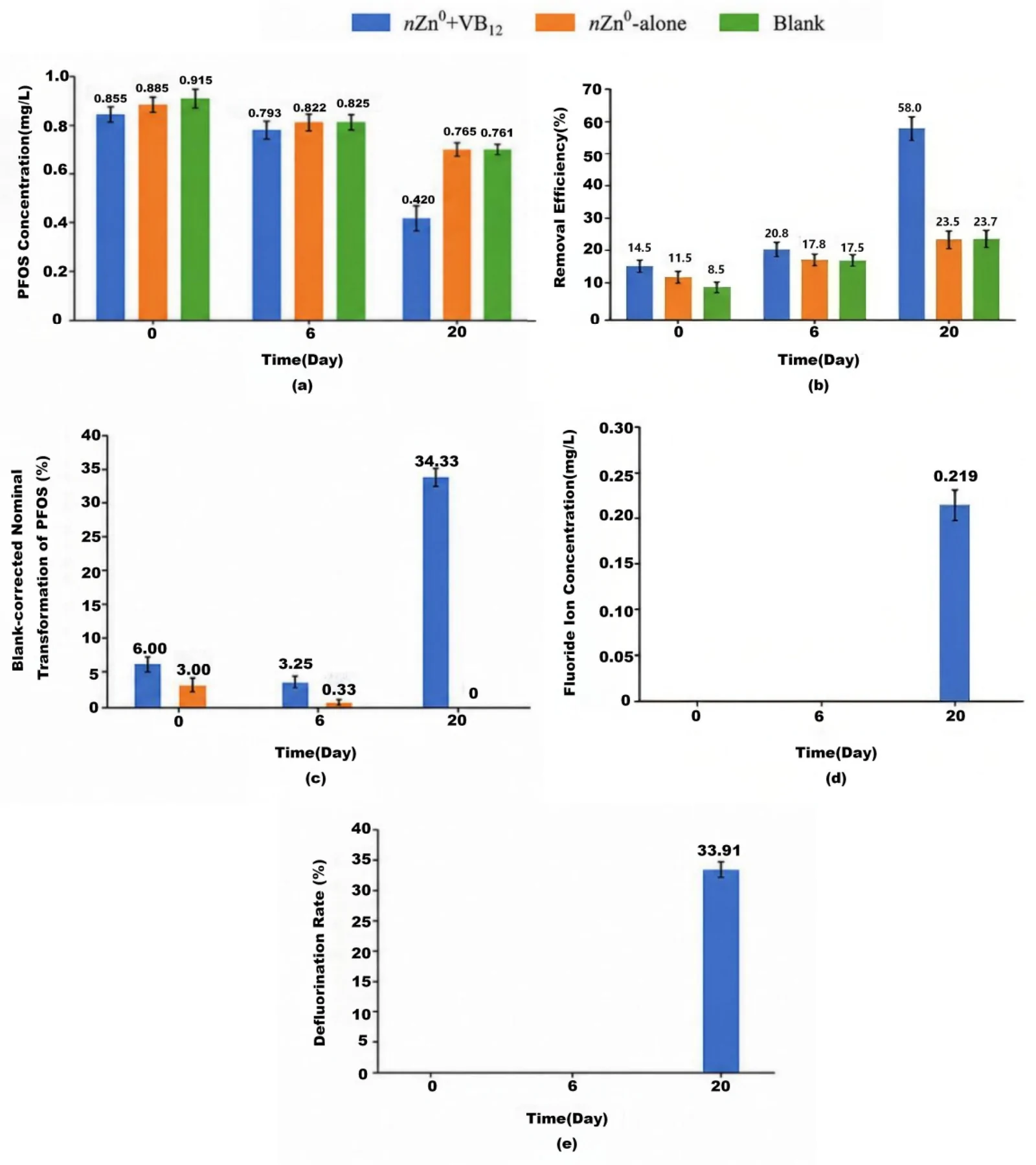
Time-dependent changes in PFOS concentration, removal, transformation, fluoride release, and defluorination rate: (**a**) change in PFOS concentration over time; (**b**) removal rate of PFOS; (**c**) blank-corrected apparent transformation of PFOS; (**d**) average fluoride ion concentration of PFOS (blank-subtracted) over time; and (**e**) defluorination rate of PFOS.

**Figure 2:**
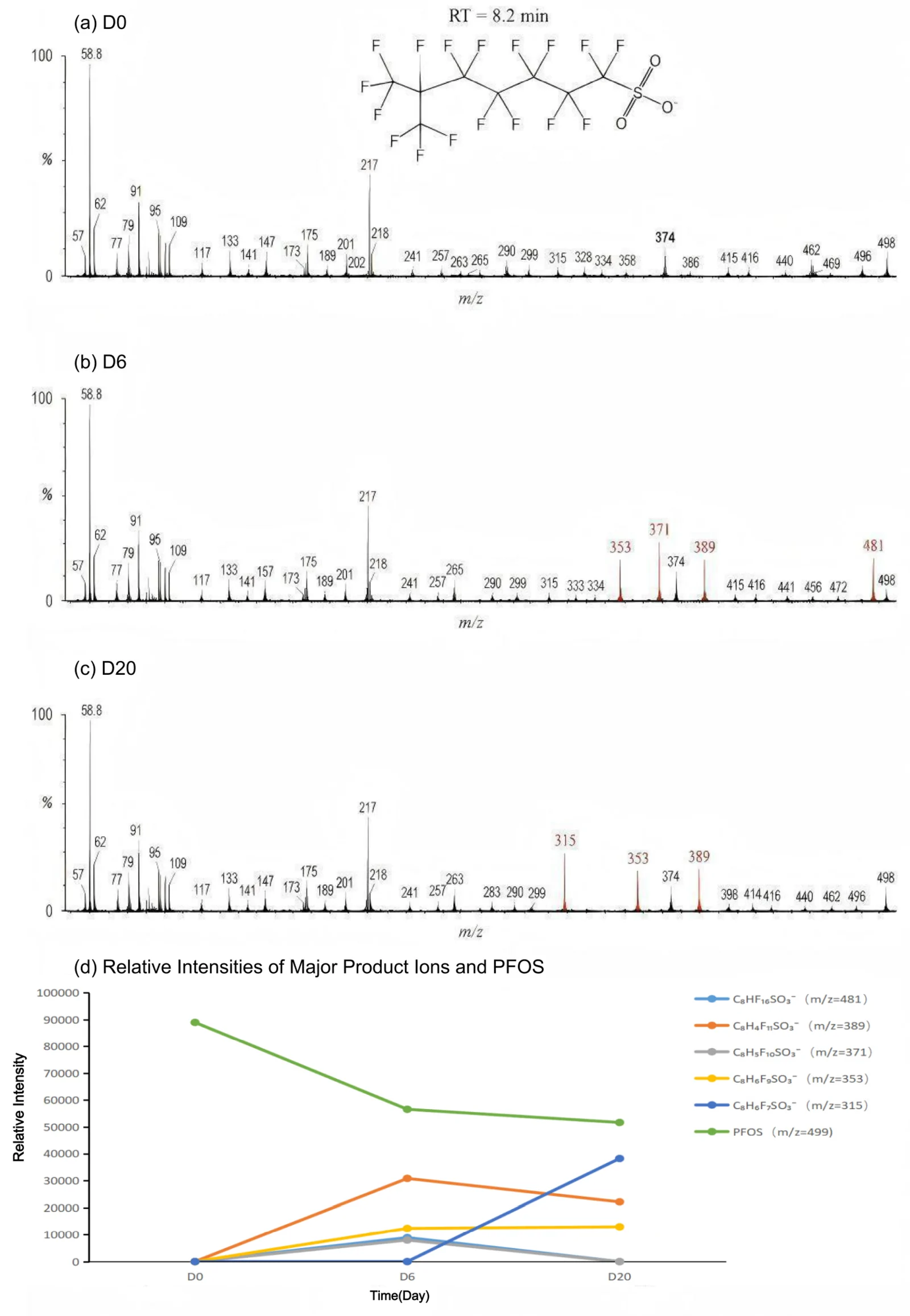
Time-dependent changes in PFOS and its tentative transformation products revealed by LC-MS: (**a**) full-scan LC-MS spectrum of PFOS at day 0 (D0); (**b**) full-scan LC-MS spectrum of PFOS and its tentative transformation products at day 6 (D6); (**c**) full-scan LC-MS spectrum of PFOS and its tentative transformation products at day 20 (D20); and (**d**) changes in the relative response intensities of PFOS (shown at 1/10 of its actual value) and the major tentative transformation products over time. Red markers in (**b**,**c**) indicate the detected tentative transformation products.

**Figure 3:**
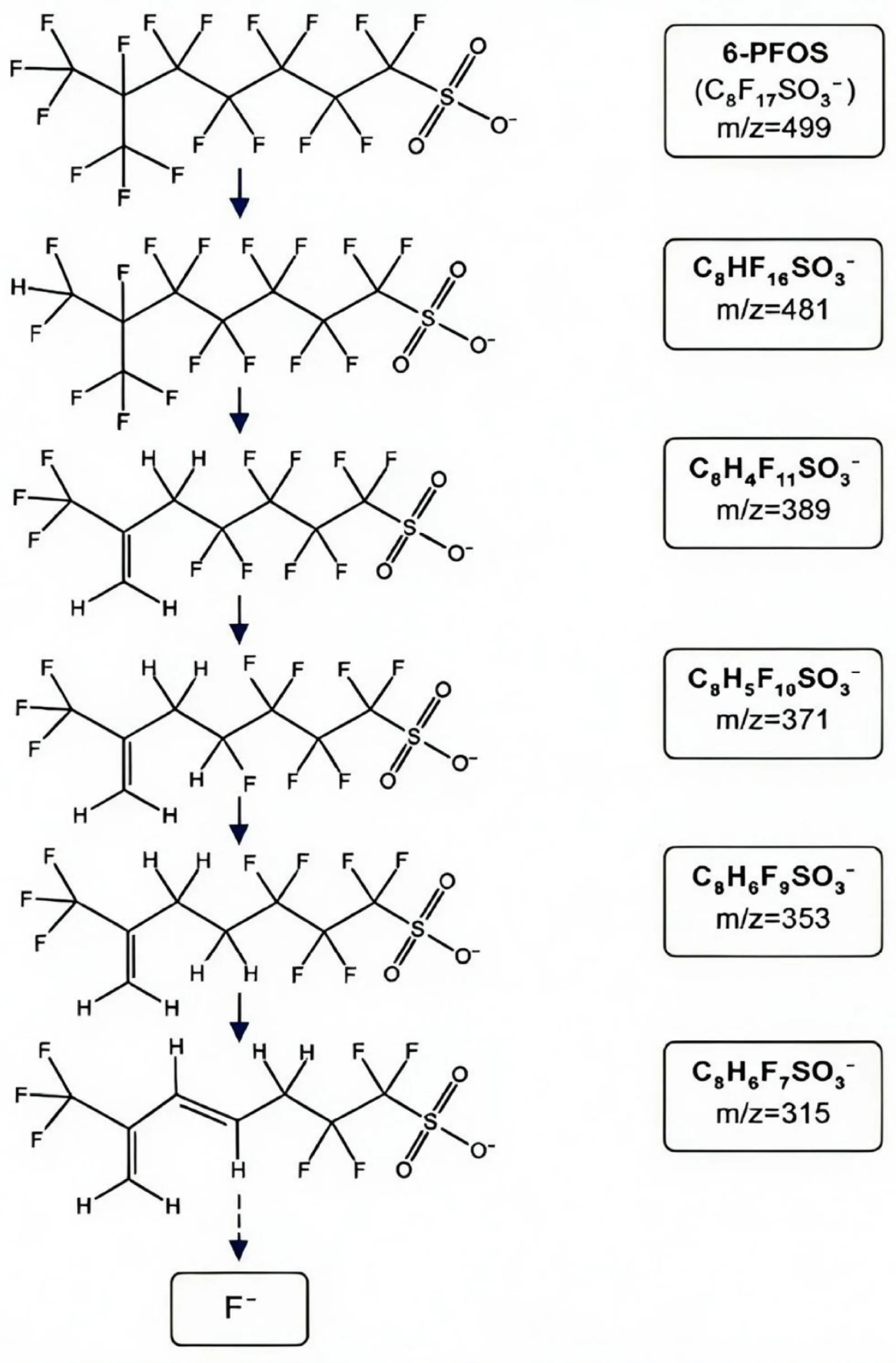
Proposed transformation pathway of PFOS in the nZn^0^ + VB12 system; product structures and the proposed pathway are tentative and require confirmation by authentic standards and high-resolution MS/MS. The solid lines represent the detected products in the pathway, and the dashed line represents the speculated product in the pathway.

**Figure 4:**
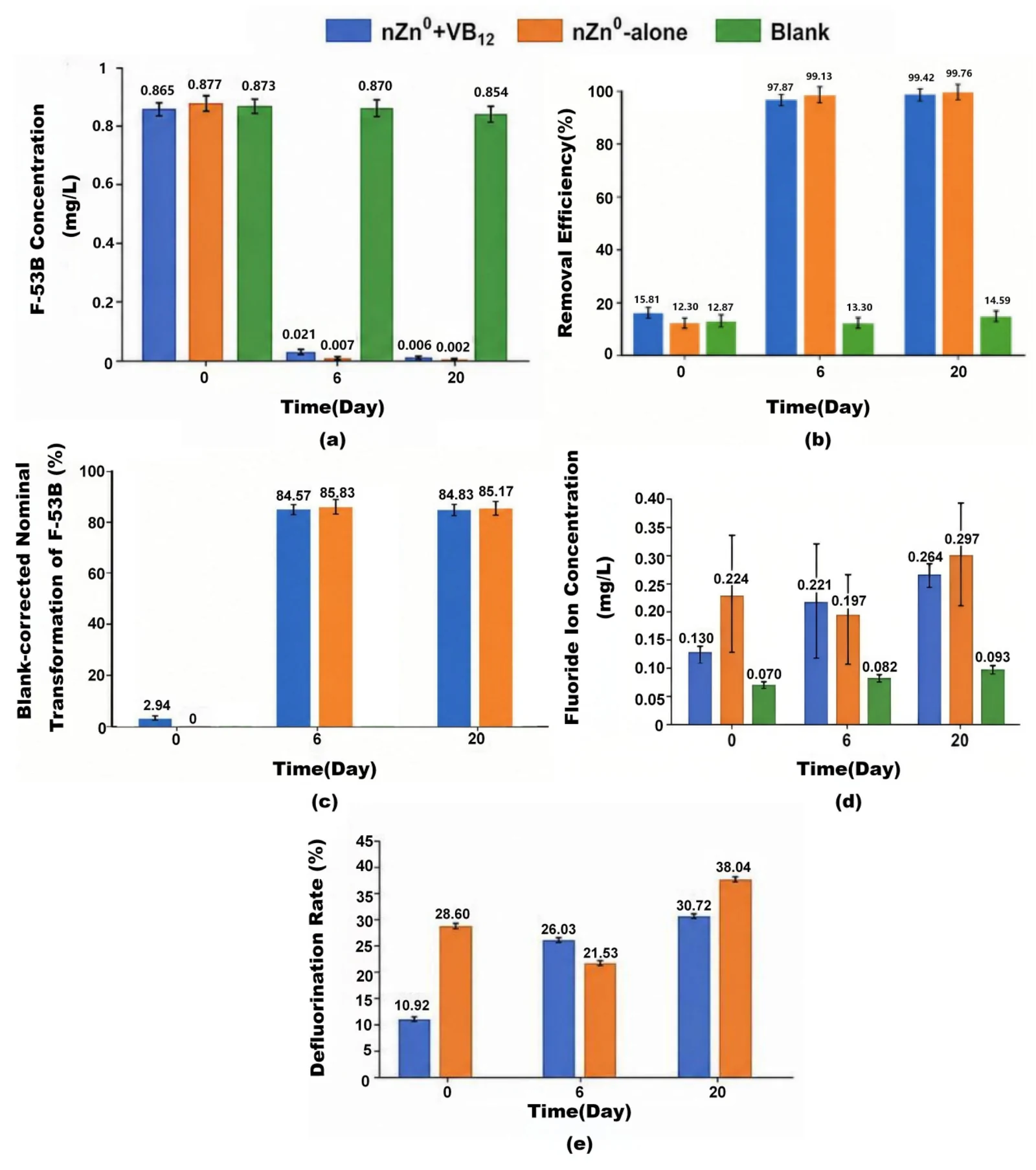
Time-dependent changes in F-53B concentration, removal, transformation, fluoride release, and defluorination rate: (**a**) change in F-53B concentration over time; (**b**) removal rate of F-53B; (**c**) blank-corrected apparent transformation of F-53B; (**d**) average fluoride ion concentration of F-53B (blank-subtracted) over time; and (**e**) defluorination rate of F-53B.

**Figure 5:**
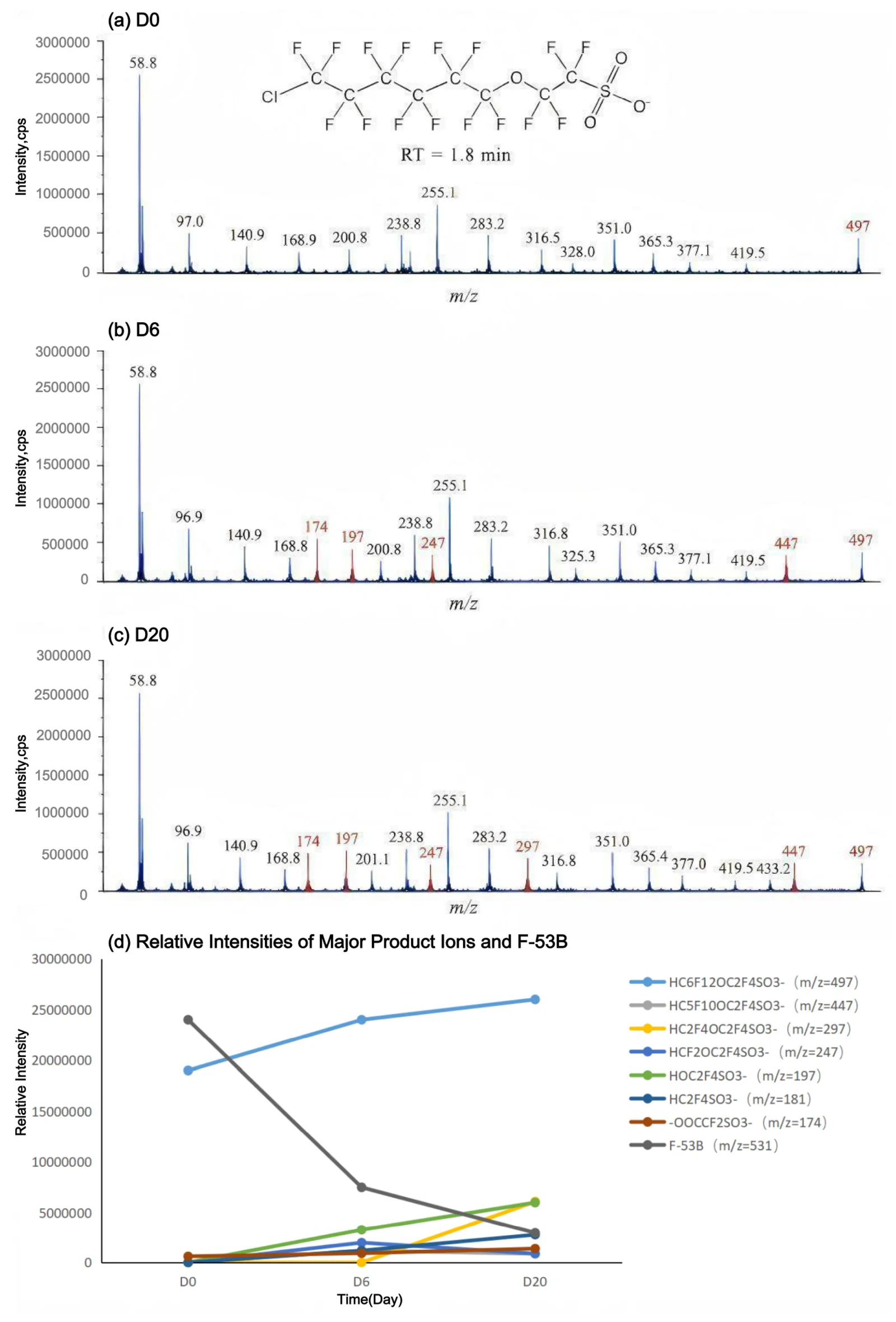
Time-dependent changes in F-53B and its tentative transformation products revealed by LC-MS: (**a**) full-scan LC-MS spectrum of F-53B at day 0 (D0); (**b**) full-scan LC-MS spectrum of F-53B and its tentative transformation products at day 6 (D6); (**c**) full-scan LC-MS spectrum of F-53B and its tentative transformation products at day 20 (D20); and (**d**) changes in the relative response intensities of F-53B and the major tentative transformation products over time. Red markers in (**a**–**c**) indicate the detected tentative transformation products.

**Figure 6:**
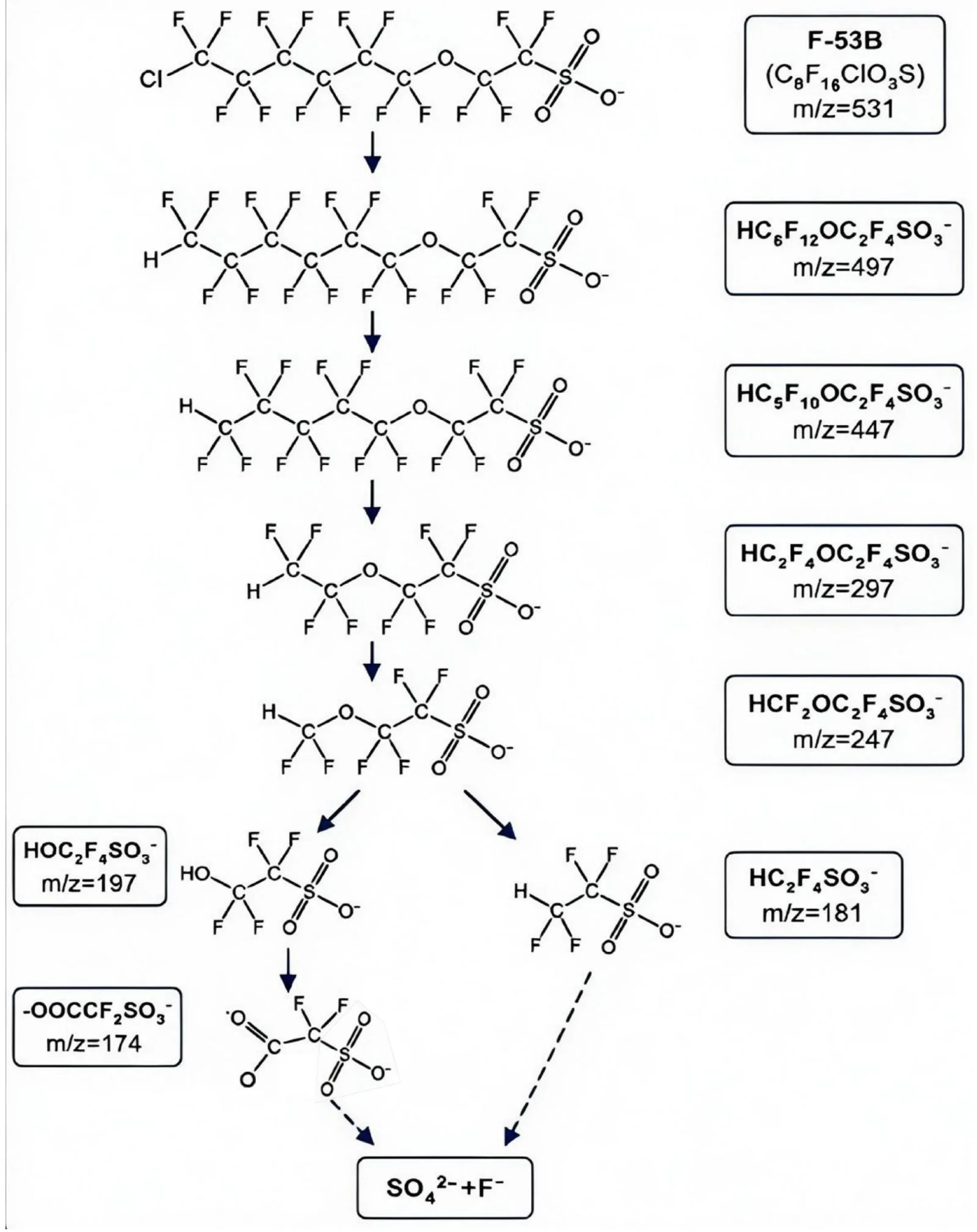
Proposed transformation pathway of F-53B in the nZn^0^-based systems; product structures and the proposed pathway are tentative and require confirmation by authentic standards and high-resolution MS/MS (the *m*/*z* 497 feature was also detected in the Blank). The solid lines represent the detected products in the pathway, and the dashed lines represent the speculated products in the pathway.

**Table 1:** Setup conditions for the experiments.

Treatment	PFOS or F-53B (mg/L)	nZn^0^ (mg/L)	VB12 (mg/L)
nZn^0^ + VB12	1	278	67.8
nZn^0^-alone	1	278	None
Blank	1	None	None

## Data Availability

The original contributions presented in this study are included in the article/Supplementary Materials. Further inquiries can be directed to the corresponding author.

## Supplementary Materials

The following supporting information can be downloaded at: https://www.mdpi.com/article/10.3390/toxics14100844/s1, Figure S1. MRM chromatograms of F-53B samples after pretreatment by solid-phase extraction (upper panel) and direct filtration (lower panel); Figure S2. Comparison of Fluoride Concentrations Measured by Ion-Selective Electrode and Ion Chromatography.
