# Supplementary material for: Effects of Vitamin B12 on the Reductive Transformation of PFOS and F-53B by Nanoscale Zero-Valent Zinc
Source: Toxics. 2026 Sep 23;14(10):844. doi: 10.3390/toxics14100844 (PMC13709694; doi:10.3390/toxics14100844)
Supplement: Supplementary file 1 [file toxics-14-00844-s001.zip › toxics-4558906-supplementary.pdf]

Supplementary Materials

Effects of Vitamin B12 on the Reductive Degradation of PFOS and F-53B by Nanoscale Zero-Valent Zinc

Shunshun Gao, Tao Wang, Yun Yang, Qingzhi Cai, Xinlei Wang, and Xiaoxia Lu*

Ministry of Education Laboratory for Earth Surface Processes, College of Urban and Environmental Sciences, Peking University, Beijing 100871, China
* Correspondence: luxx@urban.pku.edu.cn

Figure S1. MRM chromatograms of F-53B samples after pretreatment by solid-phase extraction (upper panel) and direct filtration (lower panel).

Figure S2. Comparison of Fluoride Concentrations Measured by Ion-Selective Electrode and Ion Chromatography

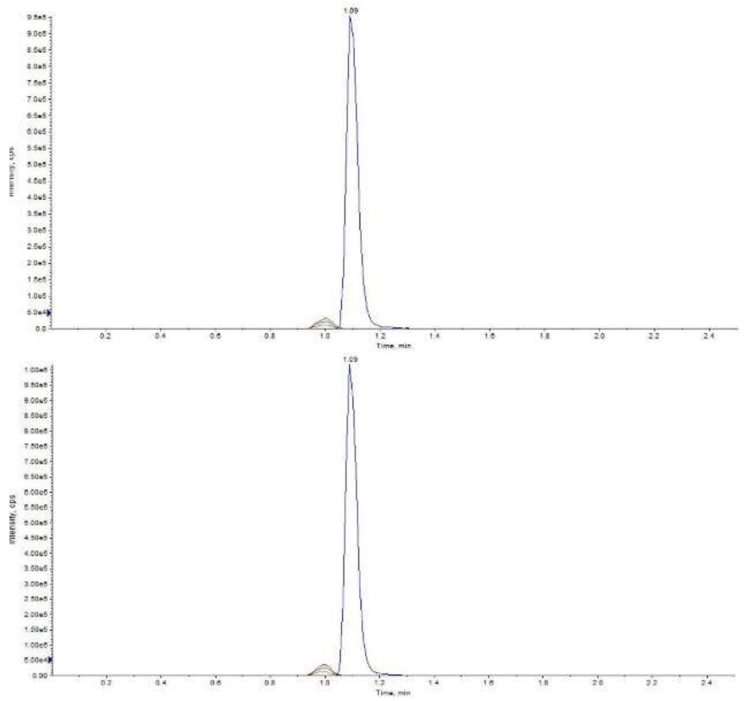

Figure S1. MRM chromatograms of F-53B samples after pretreatment by solid-phase extraction (upper panel) and direct filtration (lower panel)

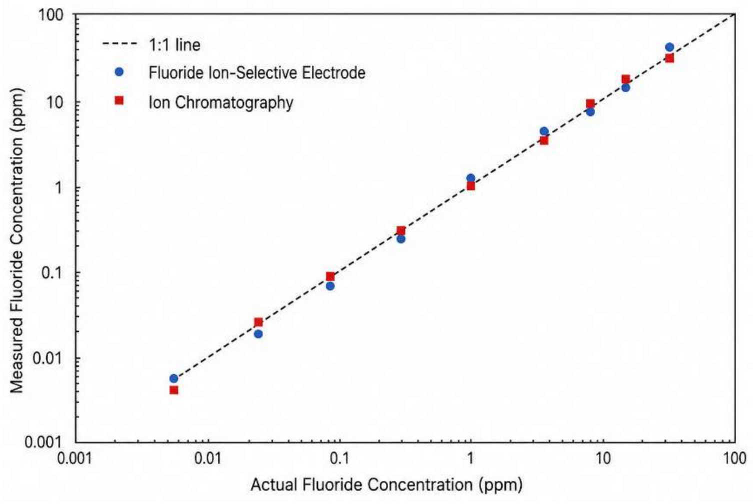

Figure S2. Comparison of Fluoride Concentrations Measured by Ion-Selective Electrode and Ion Chromatography
